# Efficacy and safety of repetitive transcranial magnetic stimulation with different frequencies on neuropathic orofacial pain: a systematic literature review and meta-analysis

**DOI:** 10.22514/jofph.2024.013

**Published:** 2024-06-12

**Authors:** Manxia Liao, Yingxiu Diao, Jiaxin Pan, Ling Shing Wong, Geetha Subramaniam, Rajkumar Krishnan Vasanthi, Linrong Liao

**Affiliations:** Rehabilitation Medicine Center, The First Dongguan Affiliated Hospital, Guangdong Medical University, 523710 Dongguan, Guangdong, China; Faculty of Health and Life Sciences, INTI International University, 71800 Nilai, NS, Malaysia; School of Rehabilitation Medicine, Gannan Medical University, 341000 Ganzhou, Jiangxi, China; Department of Rehabilitation Medicine, The Second School of Clinical Medicine, Guangdong Medical University, 518100 Dongguan, Guangdong, China; Dongguan Key Laboratory of Intelligent Rehabilitation, 523710 Dongguan, Guangdong, China

**Keywords:** Neuropathic orofacial pain, Repetitive transcranial magnetic stimulation, Rehabilitation, Meta-analysis, Neuromodulation

## Abstract

Repetitive transcranial magnetic stimulation (rTMS) is a non-invasive technique used to treat neuropathic orofacial pain (NOP). This study aimed to evaluate the efficacy and safety of rTMS in managing NOP and reducing health risks. A comprehensive literature search was conducted in various databases, including PubMed, Physiotherapy Evidence Database (PEDro), the Cochrane Library, Web of Science, Embase and Clinical Trials.gov. Thirteen relevant articles were identified and assessed using the Preferred Reporting Items for Systematic Reviews and Meta-Analyses (PRISMA) guidelines and the Cochrane Risk of Bias assessment tool. The Grading of Recommendations Assessment, Development and Evaluation (GRADE) system was utilized to evaluate the evidence rating for the studies. The analysis of the thirteen randomized controlled trials, involving 355 eligible patients, demonstrated moderate evidence supporting the significant effect of rTMS in reducing pain intensity (Mean Difference (MD): −1.01, 95% Confidence Interval (CI) −2.39 to −1.48, *p *< 0.001) and improving the quality of life (QOL) based on various instruments (MD: −9.23, 95% CI −11.91 to −6.54, *p *< 0.001; MD: −2.1, 95% CI −3.74 to −0.45, *p *< 0.05). Patients also reported favorable improvements in global impression (MD: −0.54, 95% CI −1.02 to −0.07, *p *< 0.05) and sensory status (Standardized Mean Difference (SMD): −1.30, 95% CI −1.74 to −0.87, *p *< 0.001). However, there were no significant improvements in sleep quality (MD: −1.72, 95% CI −4.13 to 0.68, *p *> 0.05) or psychological status (*p *> 0.05). Overall, the study demonstrated that rTMS is an effective and safe way to reduce pain, improve QOL, enhance sensory status, and create a positive clinical impression in patients with NOP. Further research is needed to investigate the effects of rTMS on sleep and psychological well-being in individuals with NOP.

## 1. Introduction

Neuropathic orofacial pain (NOP) is a term used to describe various disorders that cause pain in the oral, facial, head and neck areas. These conditions can range from inflammatory diseases to neuropathic pain syndromes and are among the most prevalent pain disorders [1]. Examples of neuroinflammatory pain in the oral-facial region include burning mouth syndrome (BMS), atypical facial pain (AFP), temporomandibular disorders (TMD), postherpetic neuralgia (PHN), glossopharyngeal neuralgia, idiopathic facial paralysis (IFP), and trigeminal neuralgia (TN) [2, 3]. The oral-facial region is particularly susceptible to these pain conditions, which can manifest as acute, subacute or chronic pain. Studies have shown that 19% to 26% of adults experience mouth or face pain within a month, and in some cases, this number may be as high as 48%. Chronic pain in this area affects 8% to 15% of individuals [4].

When NOP symptoms persist for more than six months, they can cause psychological distress, pain and a reduced quality of life (QOL). As a result, effective treatment for NOP can be challenging, and it can impose a significant financial burden [5, 6, 7]. Different types of NOP have distinct inflammatory processes and neuropathic pain mechanisms. Various inflammatory factors may cause neuronal damage, resulting in either neuropathic or inflammatory pain [8].

Clinicians need to have a comprehensive understanding of the pathophysiology, diagnosis and treatment options for different types of NOP diseases to ensure appropriate care [9]. The inflammatory environment can influence the effectiveness of treatment interventions. Conservative approaches are frequently used to alleviate inflammatory pain and improve symptoms. Medications such as nonsteroidal anti-inflammatory drugs (NSAIDs), gabapentin, and certain antiepileptic drugs are often considered suitable options and can be administered topically for NOP conditions. However, these medications may not always yield satisfactory results and may only provide marginal improvements accompanied by potential side effects [10].

Repetitive transcranial magnetic stimulation (rTMS) is a non-invasive, side-effect-free therapeutic approach for managing NOP symptoms [11, 12, 13]. Long-term rTMS has been demonstrated to notably enhance neurogenesis, suppress apoptosis, and regulate inflammation. However, the precise mechanisms responsible for the curative impacts of rTMS on neurological recuperation in individuals with neuropathic pain (NOP) are ambiguous [14].

The stimulation of the left prefrontal cortex is often associated with treating depression and improving mood, while stimulation of the primary motor cortex (M1) is frequently applied on the opposite side of the pain region to produce additional analgesic effects [15]. The effects of rTMS may vary depending on the stimulated area and the frequency of stimulation. For instance, 10 Hz stimulation was found to be superior to 5 Hz in terms of pain improvement, although the latter had better safety [16]. Fricová *et al*. [17] (2013) reported that rTMS can be systematically employed for the treatment of chronic pain, with the optimal therapeutic effect being dependent on determining the precise duration and intensity of each stimulation. Their pilot study concluded that 20 Hz stimulation was more effective than 10 Hz stimulation. Nonetheless, there are currently no comprehensive standards or clinical guidelines regarding the application of rTMS in patients experiencing neuropathic pain (NOP). Conducting a systematic literature review and meta-analysis of relevant randomized controlled trials (RCTs) can furnish valuable medical evidence for utilizing rTMS to address various types of NOP symptoms. Additionally, elucidating the mechanisms underlying how rTMS controls inflammatory pain while improving symptoms is necessary, alongside conducting qualitative and quantitative analyses to identify any adverse events that may arise.

## 2. Materials and methods

### 2.1 Study protocol and registration

We conducted a meta-analysis study adhering to the PICOS (Population, Intervention, Comparison, Outcome, Study design) strategy and registered it on the PROSPERO website (www.crd.york.ac.uk/PROSPERO/). The study protocol follows the guidelines set out in the Preferred Reporting Items for Systematic Reviews and Meta-Analysis (PRISMA). The registration number for this meta-analysis is CRD42022372347.

### 2.2 Types of studies

We reviewed randomized controlled trials (RCTs) published in English, excluding literature reviews, systematic reviews, meta-analyses, prospective cohort studies, retrospective studies, case series articles, and any studies with incomplete or missing information. By limiting our analysis to RCTs, we aimed to ensure a higher level of evidence and reduce bias in our findings.

### 2.3 Types of participants

This meta-analysis included adult patients aged 18 years and older who had a clinical diagnosis of neuropathic orofacial pain (NOP). This encompassed several specific conditions, including BMS, AFP, TMD, PHN, glossopharyngeal neuralgia, IFP and TN. By including these specific subtypes of NOP, we aimed to provide a comprehensive analysis of the effectiveness of repetitive transcranial magnetic stimulation (rTMS) in various forms of orofacial pain.

### 2.4 Types of interventions

We considered different types of rTMS interventions in this meta-analysis, including rTMS with different frequencies, such as theta burst stimulation (TBS) modes like intermittent TBS (iTBS) and continuous TBS (cTBS). We also included repetitive peripheral magnetic stimulation (rPMS) as an intervention. The interventions included the use of rTMS or rPMS alone, or in combination with other forms of physical therapy. The specific stimulus parameters used in these interventions, such as intensity, duration and frequency, are described and documented in the included studies. By analyzing these different types of interventions and their respective parameters, we aim to assess their therapeutic effects on various subtypes of NOP.

### 2.5 Types of outcome measures

The primary focus of this meta-analysis was to examine the intensity of pain in the orofacial region. Additionally, other outcome measures such as the sleep quality (SQ), psychological and clinical status, quality of life (QOL), and sensory status. The method parameters used in the interventions (such as the specific rTMS or rPMS protocols and parameters) were also taken into consideration.

In addition to assessing the efficacy of the interventions, any adverse reactions were also evaluated. Such reactions usually involve mild discomfort, such as pain, dizziness, or other symptoms experienced by the patients. The examination of these outcome measures enabled us to evaluate the impact of repetitive transcranial magnetic stimulation (rTMS) and repetitive peripheral magnetic stimulation (rPMS) on pain reduction, sleep quality, psychological well-being, quality of life, sensory status, and any potential adverse reactions in patients with NOP.

### 2.6 Data sources and search strategy

To carry out the meta-analysis, we searched six electronic databases: PubMed, Physiotherapy Evidence Database (PEDro), the Cochrane Library, Web of Science, Embase and Clinical Trials.gov. Two researchers (XML and JXP) followed specific inclusion criteria to retrieve relevant studies from the inception of the databases up until 10 November 2022. The search was limited to studies published in English. We used a combination of Medical Subject Headings (MeSH) words and free words to enhance the search process. The MeSH words included terms related to orofacial pain, neuropathic pain and transcranial magnetic stimulation. The free words encompassed specific conditions such as burning mouth syndrome, atypical facial pain, temporomandibular disorders, postherpetic neuralgia, glossopharyngeal neuralgia, idiopathic facial paralysis, and trigeminal neuralgia. Furthermore, a secondary search of published systematic reviews was conducted to identify any relevant studies that met the inclusion criteria. For more information, please refer to the attached document (**Supplementary material**) which provides the comprehensive search details.

### 2.7 Data extraction and management

The data extraction process was conducted by MXL and YXD, using a standardized methodology. The extracted information was organized in a pre-developed table format. Any discrepancies in the extracted data were resolved through discussion between the researchers and the corresponding authors. For each study included in the meta-analysis, we extracted the following data points: first author’s name, year of publication, country where the study was conducted, sample size, outcome measures evaluated alongside their corresponding means and standard deviations. Additionally, details such as the subclasses of NOP, patient age range, stimulation site, pain duration, type of TMS used, specific adverse events, intervention frequency and intensity, thresholds, and follow-up periods were also extracted. All collected data was subsequently imported into Microsoft Excel (version 2021, Microsoft Corporation, Redmond, WA, USA) for organization and management purposes. In cases where there was ambiguity or insufficient information provided in original articles reviewed for this analysis; we contacted corresponding authors *via* email requesting further clarification or additional information needed to complete our assessment accurately. This meticulous approach ensures that all relevant data is captured comprehensively enabling a rigorous analysis required for this meta-analysis research work.

### 2.8 Quality evaluation and rating of evidence

MXL and YXD conducted a risk assessment for each randomized controlled trial (RCT) included in the meta-analysis, following the guidelines outlined in the Cochrane Handbook for Systematic Review of Interventions [18] and utilizing the Cochrane Collaboration tool as RoB 2.0. The RoB 2.0 was employed to evaluate bias risk across five domains: randomization process, deviations from intended interventions, missing outcome data, measurement of outcomes and selection of reported result [19]. Bias risk for each domain was then classified as low, unclear or high.

Furthermore, the Grading of Recommendations Assessment Development and Evaluation (GRADE) system was used to assess evidence rating for all included studies. This approach evaluates evidence level by rating it as very low, low, medium or high [20]. Five factors that may affect quality of RCTs evidence are taken into account including publication bias; indirectness; inconsistency; imprecision and risk of bias. By using these well-established tools and methodologies we can adequately evaluate study quality and evidence level allowing us to comprehensively assess overall strength available for this meta-analysis review.

### 2.9 Data synthesis and analysis

The first author, MXL, conducted the data synthesis and analysis for this study. The raw data from the articles included in the study was summarized using data collection sheets designed for this qualitative and quantitative analysis. Statistical analysis of the outcome indicators’ data was performed using RevMan software (version 5.4, The Cochrane Collaboration, London, UK). Effect sizes for dichotomous data were reported as relative risk (RR) with 95% confidence intervals (CI), while results for continuous data were reported as standardized mean difference (SMD) or mean difference (MD), as appropriate [21]. Median and quartile data were converted to mean and standard deviation if the original studies provided them. To assess heterogeneity among the included studies, a heterogeneity test was conducted. A fixed-effect model was employed for data combination if the *I*^2^ statistic was less than or equal to 50%, indicating low heterogeneity.

However, if the *I*^2^ statistic was greater than 50%, indicating high heterogeneity, a random-effects model was used [22]. The choice of model (fixed or random effects) was based on the clinical and methodological heterogeneity observed among the studies. The *p*-value set for statistical significance was less than 0.05. To assess publication bias, a funnel plot test was conducted to evaluate the potential impact of publication bias on the overall findings. Subgroup analysis was performed to explore potential heterogeneity among the studies based on different characteristics. This analysis aimed to investigate the influence of rTMS on the effectiveness of interventions for NOP. In summary, this data synthesis and analysis approach allowed for a comprehensive evaluation of the included studies, exploration of heterogeneity, and examination of the impact of rTMS on the intervention outcomes for NOP.

## 3. Results

### 3.1 Literature selection

The process of retrieving data involved importing content from six specified databases into the Endnote X9 software, which Clarivate Analytics in London, UK developed. Initially, a total of 1983 references were retrieved, but 546 duplicates were identified and removed, which resulted in 1437 articles. A search of the references from the eligible studies was conducted, and 1418 reviews and other unrelated studies were excluded to ensure that only relevant articles were included in the subsequent analysis. After this process, only 19 articles remained and were selected for a full-text search. After careful evaluation against the predefined selection criteria, 13 RCTs [16, 17, 23, 24, 25, 26, 27, 28, 29, 30, 31, 32, 33] were deemed suitable for inclusion. A detailed flow chart of the literature selection process can be found in Fig. 1, illustrating the step-by-step progression from the initial retrieved references to the final selection of the relevant RCTs.

**Fig. 1.** PRISMA flow diagram.

### 3.2 Risk of bias and certainty of evidence

Fig. 2 provides an overview of the overall risk of bias for the thirteen RCTs that were included. Fig. 3 provides a detailed breakdown of the risk of bias assessment for each study. The risk of bias assessment results were consistent across all outcome measures; therefore, only the main outcome is presented in Fig. 3. Most of the studies demonstrated a low risk of bias concerning the randomization process. However, two studies [27, 31] raised some concerns due to their use of non-randomized study designs. For the remaining four domains (deviations from intended interventions, missing outcome data, measurement of the outcome, and selection of the reported result), all included RCTs had a low risk of bias. Overall, the risk of bias across the included studies were considered to be low to moderate.

The meta-analysis included data from four RCTs [16, 23, 26, 32], which included a total of 313 patients. The analysis estimated risk ratios for all comparisons, which were found to be above 1.0, suggesting no significant risk associated with rTMS compared to sham rTMS. The estimated risk ratio for rTMS versus sham rTMS was 1.26 (95% CI 0.74 to 2.16), indicating a moderate certainty about the evidence (Table 1). In addition, the level of evidence for the relevant clinical outcome indicators, including visual analog scale (VAS), sleep quality (SQ), QOL, Brief Pain Inventory (BPI), Patient Global Impression of Change (PGIC), and quantitative sensory testing (QST), ranged from very low to moderate. Overall, based on the risk of bias assessment and the level of evidence, the certainty of evidence for the effects of rTMS on NOP was determined to be moderate.

**Fig. 2.** Risk of bias summary. rTMS: repetitive transcranial magnetic stimulation.

**Fig. 3.** Risk of bias assessment of individual studies: no difference in the results for the outcomes assessed in each study.

**Table 1.** The grading of recommendations assessment, development, and evaluation (GRADE) system.

Outcomes	Risk of bias	Inconsistency	Indirectness	Imprecision	Other considerations	Certainty of the Evidence (GRADE)	Relative effect (95% CI)	Absolute effects (95% CI)	Importance
VAS	No serious	Serious^c^ inconsistency	No serious indirectness	Serious^b^ imprecision	Reporting bias^d^	Very low ⊕◯◯◯	-	MD 1.94 lower (2.39 to 1.48 lower)	Critical
SQ	Serious^a^	Serious^c^ inconsistency	No serious indirectness	Serious^b^ imprecision	Reporting bias	Very low ⊕◯◯◯	-	MD 1.72 lower (4.13 lower to 0.68 higher)	Critical
QOL	Serious^a^	No serious inconsistency	No serious indirectness	Serious^b^ imprecision	Reporting bias	Low ⊕⊕◯◯	-	MD 9.23 lower (11.91 to 6.54 lower)	Critical
BPI	No serious	Serious^c^ inconsistency	No serious indirectness	No serious imprecision	Reporting bias	Moderate ⊕⊕⊕◯	-	MD 2.1 lower (3.74 to 0.45 lower)	Critical
PGIC	Serious^a^	Serious^c^ inconsistency	No serious indirectness	Serious^b^ imprecision	Reporting bias^d^	Very low ⊕◯◯◯	-	MD 0.54 lower (1.02 to 0.07 lower)	Critical
QST	No serious	No serious inconsistency	No serious indirectness	Serious^b^ imprecision	Reporting bias	Moderate ⊕⊕⊕◯	-	SMD 1.3 lower (1.74 to 0.87 lower)	Critical
Adverse effects	No serious	No serious inconsistency	No serious indirectness	No serious imprecision	Reporting bias	Moderate ⊕⊕⊕◯	RR 1.26 (0.74 to 2.16)	31 more per 1000 (from 31 fewer to 140 more) 65 more per 1000 (from 65 fewer to 290 more)	Important

*^a^Downgraded by one level: absence of description of blindness and randomization; ^b^Downgraded by one level: small sample size; ^c^Downgraded by one level: heterogeneity is high; ^d^Downgraded by one level: funnel asymmetry. GRADE Working Group grades of evidence: moderate certainty (⊕⊕⊕◯): we are moderately confident in the effect estimate: the true effect is: the true effect is likely to be close to the estimate of the effect, but there is a possibility that it is substantially different; low certainty (⊕⊕◯◯): our confidence in the effect estimate is limited: the true effect may be substantially different from the estimate of the effect; very low certainty (⊕◯◯◯): we have very little confidence in the effect estimate: the true effect is likely to be substantially different from the estimate of effect. CI: confidence intervals; VAS: visual analog scale; BPI: Brief Pain Inventory; PGIC: Patient Global Impression of Change; QOL: quality of life; QST: quantitative sensory testing; MD: mean difference; SMD: standardized mean difference; SQ: sleep quality; RR: relative risk.*

### 3.3 Characteristics of eligible studies

This study analyzed a total of 13 randomized controlled trials (RCTs) for qualitative description (systematic review), and 7 out of these studies were also used for quantitative analysis (meta-analysis). The basic traits of these included studies, along with information regarding adverse events, are summarized in Table 2. Table 3 provides a comprehensive summary of the specific parameters related to transcranial magnetic stimulation used in the included studies. Lastly, Table 4 presents the details of the NOP conditions, inclusion criteria, and main results of the included studies. These tables collectively offer a concise overview of the relevant information on the characteristics of the eligible studies, serving as a valuable reference for understanding the specific details of each study included in this review.

**Table 2.** Characteristics of included studies in this conducted systematic review.

Study	Country	Study design	Duration (mon)	Age, yr (mean ± SD/mean)	Interventions	Sample size	Gender		Follow-up (d)	Outcome measures	Adverse effects
							Female	Male			
Lefaucheur *et al*. [30], 2001	France	Crossover	N/A	57.2	Active rTMS Sham rTMS	7	N/A	N/A	None	VAS	None
Lefaucheur *et al*. [29], 2004	France	Crossover	N/A	N/A	Active rTMS Sham rTMS	12	N/A	N/A	None	VAS QST	None
Khedr *et al*. [27], 2005	Egypt	Parallel	39.0 ± 31.0	51.5 ± 10.7	Active rTMS Sham rTMS	28	N/A	N/A	15	VAS LANSS	None
Hosomi *et al*. [26], 2013	Japan	Crossover	N/A	N/A	Active rTMS Sham rTMS	6	N/A	N/A	17	VAS SF-MPQ BDI	Mild
Fricová *et al*. [17], 2013	Czech Republic	Parallel	≥6	50.7	Active rTMS Sham rTMS	59	N/A	N/A	21	VAS QST	None
Lindholm *et al*. [31], 2015	Finland	Crossover	125.2	59.5 ± 9.3	Active rTMS	16	14	2	7	NPRS BDI SF-36 BPI NePIQoL	None
Ma *et al*. [32], 2015	China	Parallel	17.3 ± 24.1 15.7 ± 23.2	65.4 ± 10.5 67.3 ± 11.9	Active rTMS Sham rTMS	40	20	20	90	VAS SF-MPQ QOL SQ PGIC SDS	Mild
Umezaki *et al*. [33], 2015	USA	Parallel	63.4 ± 65.5	63.4 ± 10.8 64.4 ± 8.3	Active rTMS Sham rTMS	20	N/A	N/A	60	VAS SF-MPQ BPI PHQ-9 PGIC CGI	None
Ayache *et al*. [25], 2016	France	Crossover	N/A	50.6 ± 11.3	Active rTMS Sham rTMS	16	11	5	21	VAS	None
Andre-Obadia *et al*. [24], 2018	France	Crossover	5.0 ± 3.2 15.0 ± 5.2	N/A	Active rTMS Sham rTMS	12	9	3	14	NPRS	N/A
Kohútová *et al*. [28], 2017	Czech Republic	Parallel	≥6	55.5 ± 12.7 59.3 ± 14.9	Active rTMS Sham rTMS	19	12	7	14	VAS BDI BAI QST	Mild
Pei *et al*. [16], 2019	China	Parallel	15.7 ± 23.20 16.5 ± 20.40 17.3 ± 24.10	67.3 ± 11.9 65.9 ± 12.3 65.4 ± 10.5	Active rTMS Sham rTMS	60	30	30	15	VAS SF-MPQ QOL SQ SDS PGIC	Mild
Liu *et al*. [23], 2022	China	Parallel	5.5 ± 6.80 4.0 ± 4.40	45.9 ± 11.7 51.3 ± 15.5	Active rTMS plus routine rehabilitation	60	31	29	5	HBGS SFGS MPS	Mild

*BPI: Brief Pain Inventory; BDI: Brief Depression Inventory; CGI-I: Clinical Global Impression for Global Improvement Scale; HBGS: House-Brackmann Grading Scale; LANSS: Leeds Assessment of Neuropathic Symptoms and Signs Scale; NePIQoL: Neuropathic Pain Impact on Quality of Life; MPS: Modified Portmann Scale; N/A: Not Available; NPRS: Numerical Pain Rating Scales; PGIC: Patients’ Global Impression of Change; PHQ-9: Patient Health Questionnaire; QOL: Quality of Life; QST: Quantitative Sensory Testing; rTMS: Repetitive Transcranial Magnetic Stimulation; SD: Standard Deviation; SDS: Self-rating Depression Scale; SFGS: Sunnybrook Facial Grading System; SF-MPQ: Short-Form McGill Pain Questionnaire; SQ: Sleep Quality; VAS: Visual Analogue Scale.*

**Table 3.** Characteristics of rTMS parameters.

Study	Instrument	Frequency (Hz)	Intensity	Duty cycle (OFF:ON)	Treatment time (min/session, sessions/wk, wk)	Total pulses	Target area	Coil type
Lefaucheur *et al*. [30], 2001	Super-Rapid Magstim magnetic stimulator (Whitland, UK)	10	80% RMT	5:55	20 min/session, ten sessions	1000	M1	Figure-of-eight coil
Lefaucheur *et al*. [29], 2004	Super-Rapid Magstim magnetic stimulator (Whitland, UK)	10	80% RMT	5:55	20 min/session, single session	1000	M1	Figure-of-eight coil
Khedr *et al*. [27], 2005	Mag-Lite r25 stimulator (Dantec Medical, Skovelund, Denmark)	20	80% RMT	10:50	10 min/session, five sessions	2000	M1	Figure-of-eight coil
Hosomi *et al*. [26], 2013	Magstim Rapid stimulator, UK	5	90% RMT	N/A	Ten sessions	500	M1	Figure-of-eight coil
Fricová *et al*. [17], 2013	Magstim Super Rapid stimulator (Magstim, Whitland, UK)	20 10	95% MT	N/A	Five sessions	720	M1	N/A
Lindholm *et al*. [31], 2015	eXimia TMS stimulator (Nextim Ltd, Helsinki, Finland)	10	90% RMT	5:10	15 min/session, single session	1000	M1/S1/S2	Figure-of-eight coil
Ma *et al*. [32], 2015	Magnetic Stimulator (Yiruide CCY-III, Wuhan, China)	10	80% RMT	5:3	40 min/session, ten sessions	1500	M1	Round Coil
Umezaki *et al*. [33], 2015	A MagVenture MagPro x100 Stimulator (MagVenture, Inc.; Denmark)	10	110% RMT	5:10	15 min/session, ten sessions	3000	L-DLPFC	Figure-of-eight coil
Ayache *et al*. [25], 2016	A MagPro X100 stimulator (MagVenture (Mag2Health), Farum, Denmark)	10	90% RMT	10:20	15 min/session, three sessions	3000	M1	Figure-of-eight coil
Andre-Obadia *et al*. [24], 2018	MagPro X100, MagVenture	20	90% RMT	N/A	26 min/session, single session	1600	M1	Figure-of-eight coil
Kohútová *et al*. [28], 2017	Magstim Super Rapid stimulator (Magstim, Whitland, UK)	50	90% MT	2:8	single session	600	M1	N/A
Pei *et al*. [16], 2019	Transcranial magnetic stimulator (Yiruide CCY-III, Wuhan, Hubei, China)	5 10	80% MT	1:1.2 0.5:3	17.5 min/session, five sessions	1500	M1	N/A
Liu *et al*. [23], 2022	NTK-TMS-II transcranial magnetic stimulation instrument (Jiangxi, China)	5	80–120% RMT	6:14	20 min/session, 5 sessions/wk, 2 wk	1800	Face	Figure-of-eight coil

*L-DLPFC: Left Dorsolateral Prefrontal Cortex; M1/S1/S2: primary motor cortex (M1), primary sensory cortex (S1), and secondary somatosensory cortex (S2); MT: motor threshold; N/A: not available; RMT: resting active motor threshold.*

**Table 4.** Aim, main results, and conclusions of included studies for this systematic review.

Study	NOP condition	Aim	Inclusion criteria	Exclusion criteria	Main results	Conclusions
Lefaucheur *et al*. [30], 2001	TN (n = 7)	To investigate the effects of rTMS on pain level assessed on a 0–10 VAS from day 1 to day 12 following the rTMS session.	(1) Chronic unilateral pharmacoresistant neuropathic pain.	(1) History of seizures	Significant pain reduction immediately after the treatment maintained 8 days after.	This study shows that a transient pain relief can be induced in patients suffering from chronic neurogenic pain.
Lefaucheur *et al*. [29], 2004	TN (n = 12)	To assess the influence of pain origin, pain site, and sensory loss on rTMS efficacy.	(1) Chronic unilateral pharmacoresistant neurogenic pain.	(1) History of seizures	The percentage pain reduction was significantly greater following Active than sham rTMS, confirming that motor cortex rTMS was able to induce antalgic effects.	Motor cortex rTMS was found to result in a significant but transient relief of chronic pain, influenced by pain origin and pain site.
Khedr *et al*. [27], 2005	TN (n = 28)	To investigate whether five consecutive days of rTMS would lead to longer lasting pain relief in unilateral chronic intractable neuropathic pain.	(1) The diagnosis of trigeminal neuralgia was based on the criteria of the International; (2) Association for the Study of Pain.	(1) Intracranial metallic devices or with pacemakers or any other device; (2) Extensive myocardial ischaemia. (3) Epilepsy.	Active-rTMS led to a greater improvement in scales than sham-rTMS, evident even two weeks after the end of the treatment.	Five daily sessions of rTMS over motor cortex can produce long-lasting pain relief in patients with trigeminal neuralgia.
Hosomi *et al*. [26], 2013	TN (n = 6)	To assess the efficacy and safety of 10 daily rTMS in patients with neuropathic pain.	(1) Meet the criteria for neuropathic pain; (2) Pain lasted 6 months or longer despite adequate treatments.	(1) Inability to write the questionnaires; (2) Dementia, aphasia, major psychiatric disease, suicidal wish, pregnancy; (3) Contraindications to TMS, like implantation of a cardiac pacemaker.	Significant immediate pain reduction, but cumulative effects not available for the face.	High-frequency rTMS of M1 is tolerable and transiently provides modest pain relief in patients with neuropathic pain.
Fricová *et al*. [17], 2013	TN (n = 17) AFP (n = 6)	To demonstrate the effectiveness of 20 Hz rTMS application in the treatment of patients with chronic orofacial pain syndrome. To compare the effectiveness of treatment relative to placebo rTMS.	(1) Orofacial pain syndrome, intractable pharmacoresistant pain; (2) Stable analgesic medication for at least 1 month before the start of the study and throughout its course and during follow-up evaluation two weeks after completion of rTMS; (3) 18–65 years of age.	(1) Severe organic brain damage or other serious diseases; (2) Which could interfere with rTMS (epilepsy); (3) Any metallic implants in the body (restrictions similar to those for an magnetic resonance imaging).	Significant pain reduction and mechanical thresholds at the end of treatment maintained 2 weeks after.	The better results with the relief of orofacial pain were obtained with 20 Hz stimulation if compared with 10 Hz stimulation.
Lindholm *et al*. [31], 2015	TN (n = 7) AFP (n = 4) BMS (n = 5)	To examine the effects of rTMS in neuropathic orofacial pain, and compared 2 cortical targets against placebo.	(1) Chronic daily neuropathic pain 4 in severity using NPRS of 0 to 10; (2) Patients had no history of seizure, pacemaker implantation, major stroke, or other contraindication for TMS.	(1) Multiple ischemic lesions and another after the pain diary follow-up because of average pain less than 4 on the NPRS Major depression.	Significant pain reduction and lower BPI scores after S2/M1 stimulation.	The right S2 cortex is a promising new target for the treatment of neuropathic orofacial pain with high-frequency rTMS.
Ma *et al*. [32], 2015	PHN (n = 40)	To investigated the efficacy of high-frequency rTMS in patients with PHN.	(1) Patients with chronic pain, moderate to severe in intensity (VAS ≥4) despite optimized pharmacological treatment; (2) Pain lasting longer than 1 month.	(1) Inability to participate in the questionnaires; (2) The presence of suicidal ideation and the presence of contraindications for rTMS.	The Active rTMS group demonstrated greater reduction of VAS than the sham group.	The results suggest that rTMS is an effective and safe therapy in patients with PHN.
Umezaki *et al*. [33], 2015	BMS (n = 20)	The aim of this randomized, controlled, single-blind study was to assess the efficacy of prefrontal rTMS for BMS.	(1) Diagnosed as having BMS daily and deep bilateral burning sensation of the oral mucosa; (2) Burning sensation for at least 4–6 months, constant intensity or increasing intensity during the day.	(1) Inflammation or autoimmune disease; (2) Major depression or personality major a history of disorders or substance abuse (except caffeine or nicotine).	Significant pain reduction immediately after the treatment maintained 60 days after treatment start. No reduction in psychosocial scores.	BMS pain was significantly improved with 2 weeks of treatment of high frequency rTMS over left DLPFC compared to sham stimulation.
Ayache *et al*. [25], 2016	TN (n = 9) AFP (n = 7)	To compare the analgesic efficacy of rTMS targeting the hand motor hot spot (non-navigated procedure) or the M1 representation of the pain region (navigated procedure).	(1) These patients had no contraindications to magnetic stimulation, including no history of epilepsy and/or ferromagnetic implant.	(1) Epilepsy; (2) Ferromagnetic implant.	Pain location influenced the results: upper or lower limb pain was significantly relieved, but not facial or hemibody pain.	Navigation may improve rTMS efficacy in patients with limb pain, whereas targeting remains to be optimized for more diffuse or facial pain.
Andre-Obadia *et al*. [24], 2018	TN (n = 12)	To compare the pain-relieving effects of motor rTMS when it was addressed to the corresponding cortical region (hand or face), or away from it.	(1) These patients had no contraindications to magnetic stimulation, including no history of epilepsy, addiction, migraine, intracranial ferromagnetic material or implanted stimulator (intracerebral or not, such as pacemaker).	(1) Epilepsy, addiction, migraine, intracranial ferromagnetic material or implanted stimulator (intracerebral or not, such as pacemaker).	Significant pain reduction with M1 hand stimulation but not with M1 face.	The results do not support a somatotopic effect of motor rTMS for neuropathic pain.
Kohútová *et al*. [28], 2017	COP (n = 19)	The aim of our double blind, sham-controlled, parallel-group, randomized study was to assess an efficacy of intermittent TBS (iTBS) in the treatment of patients with COP.	(1) Orofacial pain syndrome in the duration of at least 6 months, intractable pharmacotherapy-resistant pain; (2) 18–65 years of age;	(1) Severe organic brain damage or other serious diseases.	Significant modest pain reduction after the treatment but not 2 weeks after.	Our findings demonstrate that iTBS of M1 transiently provides transient and modest subjective pain relief in COP.
Pei *et al*. [16], 2019	PHN (n = 60)	This study aimed to observe the efficacy and safety of rTMS at different high frequencies (5 Hz, 10 Hz) for PHN.	(1) Aged above 50 years old; (2) Conforming to the diagnostic criteria of PHN, PHN lasting for over one month. VAS above 4, and having clear consciousness.	(1) Personal or family history of epilepsy; (2) History of craniocerebral surgery; (3) Intracranial implants; (4) Cardiac pacemakers; (5) Heart, liver, or kidney insufficiency and coagulation disorders.	VAS scores in the 10-Hz rTMS group at were significantly lower compared with the 5-Hz rTMS group.	Both 5-Hz rTMS and 10-Hz rTMS are safe and effective for PHN, as they can relieve pain and improve patients’ quality of life.
Liu *et al*. [23], 2022	IFP (n = 60)	The purpose of this study was to evaluate the clinical efficacy of peripheral rTMS in the treatment of IFP.	(1) Bell’s palsy (idiopathic facial neuritis) was diagnosed and the facial nerve was damaged identified by electromyography; (2) Initially onset and unilateral facial paralysis; (3) Aged between 18 and 75; (4) Onset was within a month, and the grading of HBGS was not less than 3 level; (5) Sign informed consent voluntarily.	(1) Patients with central facial paralysis; (2) IFP caused by Lyme disease, encephalitis, tumor or trauma and other reasons; (3) Delirious and unable to cooperate with the treatment of patients; (4) Contraindications of rTMS therapy such as pregnancy or intracranial metal foreign body, history of epilepsy, and implantation of pacemaker; (5) Patients with poor treatment compliance.	After a 2-week intervention, HBGS, SFGS, and MPS increased in both groups.	rTMS is a safe and effective noninvasive method for the treatment of idiopathic facial paralysis.

*AFP: Atypical facial pain; BPI: Brief Pain Inventory; BMS: Burning mouth syndrome; COP: chronic orofacial pain; HBGS: House-Brackmann Grading Scale; M1/S1/S2: primary motor cortex (M1), primary sensory cortex (S1), and secondary somatosensory cortex (S2); iTBS: intermittent theta burst stimulation; MPS: Modified Portmann Scale; NPRS: Numerical Pain Rating Scales; PHN: Postherpetic Neuralgia; rTMS: Repetitive Transcranial Magnetic Stimulation; SFGS: Sunnybrook Facial Grading System; TN: Trigeminal neuralgia; VAS: Visual Analogue Scale; IFP: Idiopathic Facial Palsy.*

#### 3.3.1 Participants

The 13 randomized controlled trials (RCTs) included participants from various countries, with four RCTs conducted in France [24, 25, 29, 30], three RCTs conducted in China [16, 23, 32], and two RCTs conducted in the Czech Republic [17, 28]. One study each was conducted in Egypt [27], Finland [26], the United States [31] and Japan [33]. These RCTs were published from 2001 to 2022. The sample sizes of the included studies varied from 6 to 60 participants. Of the 13 RCTs, seven were parallel controlled studies [16, 17, 23, 27, 28, 32, 33], while the remaining six were crossover trials [24, 25, 26, 29, 30, 31]. In total, 355 participants were involved in the included studies.

To simplify the analysis, the studies were categorized based on different types of NOP conditions. Among the included participants, 98 were diagnosed with TN, 17 with AFP, 25 with BMS, 100 with PHN and 60 with IFP. Additionally, 55 patients did not report specific classifications for their NOP conditions.

#### 3.3.2 Interventions

In the review, 12 studies compared active rTMS to sham rTMS [16, 17, 24, 25, 26, 27, 28, 29, 30, 31, 32, 33], while one of them also used the theta burst stimulation (TBS) model [28]. Another study [23] combined rPMS with conventional rehabilitation methods, such as acupuncture and medication. The parameters of rTMS used in the studies for treating NOP patients were as follows: the frequency ranged from 5 Hz to 50 Hz, the resting motor threshold (RMT) ranged from 80% to 120%, and the total number of pulses ranged from 500 to 3000. The treatment duration varied from 15 to 26 minutes, and the number of intervention sessions ranged from 1 to 10. Stimulation at the M1 site was mentioned in 11 studies [16, 17, 24, 25, 26, 27, 28, 29, 30, 31, 32], while one study (Umezaki *et al*. [33], 2016) mentioned stimulation at the dorsolateral prefrontal cortex (DLPFC). Only one study [23] utilized peripheral stimulation targeting the face. Nine studies used a figure-of-eight coil [23, 24, 25, 26, 27, 29, 30, 31, 33], one study used a round coil [32], and the remaining studies did not mention the type of coil used [16, 17, 28]. The ratio of off-stimulation duration to on-stimulation duration (OFF/ON) for rTMS was recorded in each study and can be found in Table 3.

#### 3.3.3 Outcome measures

The studies included in the analysis used various outcome measures to evaluate different aspects of patients’ conditions such as pain, sleep quality, QOL, clinical status, sensory status and psychological status. More information about these outcome measures can be found in Table 2. Among the studies, the Visual Analog Scale (VAS) was used in 10 studies [16, 17, 25, 26, 27, 28, 29, 30, 32, 33] and the Numeric Pain Rating Scale (NPRS) was used in 2 studies [24, 31] to assess pain levels. Furthermore, four studies [16, 26, 32, 33] employed the Short-Form McGill Pain Questionnaire (SF-MPQ) to assess pain. Two studies [16, 32] used sleep quality questionnaires to evaluate sleep quality (SQ). Three studies [17, 28, 29] utilized Quantitative Sensory Testing (QST) to measure sensory status. The Patient Global Impression of Change (PGIC) was used in three studies [16, 32, 33] to assess clinical status. Quality of life was evaluated through different measures such as QOL questionnaires [16, 31, 32], the Short Form-36 (SF-36) questionnaire [31], and the BPI (Lindholm *et al*. [31], 2015; Umezaki *et al*. [33], 2016). Psychological status was assessed in two studies [16, 32] using the Self-Rating Depression Scale (SDS), in one study [28] using the Beck Anxiety Inventory (BAI), in three studies [26, 28, 31] using the Beck Depression Inventory (BDI), and in one study [33] using the Patient Health Questionnaire-9 (PHQ-9).

### 3.4 Effectiveness

Based on our analysis of randomized controlled trials (RCTs), rTMS has shown promising outcomes in the treatment of patients with neuropathic pain (NOP) when compared to sham rTMS. The treatment has been found to effectively reduce pain intensity, improve clinical status and sensory status, and enhance QOL. However, it has not significantly improved sleep quality and psychological status. Detailed information regarding the various outcome measures is as follows:

#### 3.4.1 Effectiveness of rTMS on pain intensity

The analysis shows that pain intensity was evaluated in six studies [16, 17, 28, 31, 32, 33] using the Visual Analog Scale (VAS) and involving 175 subjects in both the rTMS and control groups. The results suggest that rTMS is effective in reducing pain intensity in individuals with NOP, although the evidence is very low (mean difference (MD) = −1.01, 95% confidence interval (CI) −2.39, −1.48, *I*^2^ = 0%, *p *< 0.001, Fig. 4). Moreover, six additional studies [23, 24, 25, 26, 27, 29, 30] consistently demonstrate the positive effects of rTMS in treating pain. Therefore, based on our comprehensive analysis, it can be concluded that rTMS is effective in reducing pain intensity in individuals with NOP.

**Fig. 4.** Meta-analysis of rTMS on pain intensity measured with VAS. SD: Standard Deviation; CI: Confidence Interval.

#### 3.4.2 Effectiveness of rTMS on sleep quality

The sleep quality was evaluated in only two studies [16, 32] using sleep quality questionnaires and involving a total of 120 subjects in both the rTMS and control groups. The analysis reveals that there is very low evidence to suggest that rTMS improves sleep quality compared to sham rTMS (mean difference (MD) = −1.72, 95% confidence interval (CI) −4.13, 0.68, *I*^2^ = 94%, *p *< 0.16, Fig. 5). However, it is important to note that more robust research is needed to fully understand the impact of rTMS on sleep quality in NOP patients, as the current evidence is limited and conflicting.

**Fig. 5.** Meta-analysis of rTMS on sleep quality measured with SQ. SD: Standard Deviation; CI: Confidence Interval.

#### 3.4.3 Effectiveness of rTMS on quality of life

The QOL was assessed using QOL questionnaires and the Brief Pain Inventory (BPI) and a total of 172 samples were analyzed. The results indicate a low to moderate level of evidence suggesting that rTMS is effective in improving the QOL of NOP patients. Specifically, the analysis shows a statistically significant improvement in QOL with rTMS compared to sham rTMS (mean difference (MD) = −9.23, 95% confidence interval (CI) −11.91, −6.54, *I*^2^ = 34%, *p *< 0.001, Fig. 6). This indicates that rTMS has a positive effect in enhancing the overall QOL of individuals with NOP. Additionally, there was a significant improvement in the BPI scores with rTMS treatment compared to the control group (MD = −2.1, 95% CI −3.74, −0.45, *I*^2^ = 0%, *p *= 0.01, Fig. 6), further supporting the notion that rTMS can effectively enhance the QOL of NOP patients. Based on the analysis results, it can be concluded that rTMS has a significant positive effect on improving the QOL of individuals with NOP.

**Fig. 6.** Meta-analysis of rTMS on QOL. (A) Quality of Life; (B) Brief Pain Inventory. SD: Standard Deviation; CI: Confidence Interval; QOL: Quality of Life; BPI: Brief Pain Inventory.

#### 3.4.4 Effectiveness of rTMS on clinical status

The clinical status of patients was assessed using the PGIC scale, and data from 140 enrolled patients were analyzed. The results indicate a very low level of evidence suggesting that rTMS has a positive effect on the clinical status of patients with NOP. Specifically, the analysis shows a statistically significant improvement in the clinical status of patients who received rTMS compared to the control group (mean difference (MD) = −0.54, 95% confidence interval (CI) −1.02, −0.07, *I*^2^ = 89%, *p* = 0.02, Fig. 7), indicating that rTMS can contribute to the improvement of the clinical status of NOP patients to a certain extent. However, it is important to note that additional high-quality studies are necessary to further evaluate and confirm these findings.

**Fig. 7.** Meta-analysis of rTMS on clinical status measured with PGIC. SD: Standard Deviation; CI: Confidence Interval.

#### 3.4.5 Effectiveness of rTMS on sensory status

The study aimed to assess the effect of rTMS treatment on the sensory status of patients with neuropathic pain (NOP), using QST. The analysis was conducted on data from 55 patients. Results showed that rTMS treatment had a positive therapeutic effect on both thermal and tactile perception, leading to an overall improvement in sensory status. The analysis revealed a statistically significant difference between the rTMS treatment group and the control group (standardized mean difference (SMD) = −1.30, 95% confidence interval (CI) −1.74, −0.87, *I*^2^ = 0%, *p *< 0.001, Fig. 8), indicating that rTMS treatment is effective in addressing sensory perception changes in NOP patients. However, the quality of evidence supporting this finding is considered moderate, and further research is needed to better understand the extent and mechanisms of sensory improvement with rTMS in NOP patients.

**Fig. 8.** Meta-analysis of rTMS on sensory status measured with QST. SD: Standard Deviation; CI: Confidence Interval.

#### 3.4.6 Effectiveness of rTMS on psychological status

Various questionnaires, including the SDS [16, 32], BAI [28], BDI [26, 28, 31] and PHQ-9 [33], were used to assess the psychological status of patients with NOP. However, quantitative analysis was not possible due to the unavailability of data, which is why the findings are presented qualitatively. Based on the available studies, it was found that the use of rTMS did not improve anxiety and depression in NOP patients. The scores on these outcome measures did not show a positive response to rTMS treatment (*p *> 0.05). Therefore, it appears that rTMS does not have a favorable therapeutic effect on the psychological status of NOP patients. However, it is important to note that the lack of quantitative analysis and the limited number of studies conducted on psychological status make it difficult to draw firm conclusions. Further research is needed to explore the potential impact of rTMS on the psychological well-being of NOP patients and to better understand the therapeutic effects in this domain.

#### 3.4.7 Adverse effects

Among the included studies, adverse events associated with rTMS was reported. Our findings can be summarized as follows: No Adverse Events—A total of seven studies (53.8%) [17, 25, 27, 29, 30, 31, 33] found no evidence of any adverse events linked to rTMS therapy. Mild Discomfort: Five studies (38.5%) [16, 23, 26, 28, 32] reported instances where patients experienced mild discomfort during the course of their rTMS procedure. Higher Incidence of Adverse Events—Only one study [24] documented any significant adverse effects related to rTMS therapy; however, this study showed a higher incidence rate among those who received active rTMS treatments without heterogeneity (Relative Risk (RR) = 1.26, *I*^2^ = 0%). However, the observed differences were not statistically significant (95% CI: 0.74, 2.16; *p *> 0.05, Fig. 9). The evidence quality supporting this finding is considered moderate.

**Fig. 9.** Meta-analysis of rTMS on adverse effects. SD: Standard Deviation; CI: Confidence Interval.

## 4. Discussion

### 4.1 Summary of main results

The systematic review aimed to evaluate the effectiveness of rTMS therapy in reducing symptoms in patients with NOP. The results of the quantitative analysis and discussion revealed the following key findings: Efficacy: The analysis demonstrated that rTMS has a positive therapeutic impact on the clinical status and sensory status of NOP patients. Evidence showed that rTMS treatment improved clinical status and sensory perception. However, the evidence for the positive impact on clinical status was of very low quality, while the evidence for sensory improvement was considered moderate. Psychological Status: According to the available qualitative data, there were no significant improvements in the anxiety and depression scores of NOP patients treated with rTMS. Further research is needed in this area to draw more definitive conclusions. Adverse Events: The majority of included studies reported no adverse events associated with rTMS treatment, with only mild discomfort reported in some cases. The quality of evidence supporting adverse event analysis was considered moderate.

### 4.2 The potential mechanisms and parameters of rTMS on patients with NOP

The effectiveness of rTMS in treating NOP patients is influenced by various stimulation parameters, including the target cerebral cortex regions, stimulation intensity and frequency, and the number of pulses delivered. While the mechanisms underlying rTMS-induced pain control effects on the motor cortex are not yet fully understood, non-invasive rTMS stimulation provides insights into these analgesic mechanisms. Previous systematic reviews [34, 35] have shown that rTMS has short-term or long-term analgesic effects in NOP patients due to changes in plasticity within the central nervous system at the level of structures involved in pain production or regulation. This current study aligns with previous research conclusions regarding its effectiveness and safety to a certain extent; however, differences in individual characteristics and treatment protocols may result in varying observations of response indicators.

Many NOP conditions involve inflammation in oral-facial tissues, ranging from acute toothache and mucositis to chronic temporomandibular joint disorders (TMD) [36]. Inflammatory pain caused by trigeminal neuritis and herpes zoster can lead to physical and psychological distress in patients. rTMS stimulates receptors within the cerebral cortex transmitting signals through nerve fibers into the nervous system prompting increased blood circulation alleviating small artery spasms around the brain while reducing tension in blood vessel walls further improving inflammatory symptoms for patients with trigeminal neuralgia [24, 26]. The specific mechanisms underlying its effectiveness remain an ongoing area of investigation as well as optimizing treatments for NOP patients. Several studies provide evidence supporting positive effects on psychological status and sleep quality among NOP patients treated with rTMS [37, 38, 39, 40, 41].

For instance, Hanna *et al*. [37] (2019) conducted a study to analyze the effects of magnetic stimulation treatment on patients with trigeminal neuralgia. The study used a protocol of 10 Hz, 80% motor threshold, and 2000 pulses administered across 32 sessions over 7 weeks [37]. Their findings showed that the treatment resulted in improvements in pain and depression levels. The longer duration of the treatment and follow-up may have contributed to these positive results. Different studies vary not only in the number of stimuli and cortical targets used but also in the frequency of pulse transmission. Additionally, significant differences exist in patient diagnoses, contributing to the heterogeneity observed in this field of research. Although psychological status and sleep quality were not significant outcomes in this study, this may be because quantitative analysis may not effectively capture the subjective impact of psychological factors. On the other hand, the heterogeneity in sleep quality results could be due to variations in stimulation intensity and treatment protocols. The most commonly used stimulation intensity was 10 Hz, and the primary target area was the M1 cortex. Another study by Liu *et al*. [23] (2022) explored the use of peripheral interventions in the treatment of atypical facial neuritis and observed significant effects. This approach warrants further investigation in future interventions. The mechanism of rTMS treatment for this condition may involve improving local blood circulation in the damaged facial nerve *via* peripheral magnetic field stimulation, resulting in restored facial nerve function and subsequent symptom relief [42].

This study did not find significant results regarding the psychological status and sleep quality of patients with NOP treated with rTMS. However, other studies have reported positive effects, which may be due to the varying methods used in rTMS studies, including differences in stimulation parameters and patient profiles. Further research is needed to better understand these effects and optimize rTMS interventions for psychological and sleep-related symptoms in NOP patients. This analysis used multiple RCTs to evaluate the effectiveness of different frequencies of rTMS in patients with various types of NOP. Currently, there is a lack of consensus regarding the specific rTMS protocol for pain management in NOP patients. Nonetheless, it is essential to standardize result reporting, minimize bias risks and enhance study quality to facilitate better comparisons and identify optimal stimulation parameters and sites. Based on the comprehensive analysis of multiple outcome measures, rTMS appears to be a safe and effective treatment for various types of NOP. However, the underlying mechanism behind rTMS’s effectiveness in treating NOP remains elusive with varying durations of analgesic effects observed.

In summary, this analysis concludes that rTMS significantly improves pain levels, QOL, sensory status as well as overall impression among NOP patients. However, its impact on psychological and sleep-related symptoms appears limited. There is a pressing need for further research aimed at gaining deeper insights into the mechanisms involved in rTMS-induced pain relief alongside exploring ways to optimize its efficacy in enhancing psychological and sleep outcomes for NOP patients.

### 4.3 Strengths and limitations

This meta-analysis is the first to evaluate the effectiveness of rTMS in treating symptoms such as pain, sensory status, quality of life, and psychological status in patients with NOP. It conducts a thorough evaluation of numerous relevant to NOP patients. The analysis includes a discussion of the psychological factors contribute to patients’ pain, emphasizing the importance of taking these factors into account when treating NOP. The safety of rTMS in the treatment of NOP is investigated, yielding useful information about the safety profile of rTMS interventions. Most of the studies included in the analysis have a relatively low to moderate risk of bias, enhancing the reliability of the findings and supporting the effectiveness of rTMS in treating NOP.

However, some studies included in the analysis had mixed patient populations. This could introduce heterogeneity in the findings. Additionally, the sample size in some studies was relatively small, which might limit the generalizability of the results. The analysis only included English-language studies, which may have introduced bias by excluding relevant studies published in other languages. The included studies had short follow-up periods, making it difficult to evaluate the quality of the treatment program and device in the intervention and control groups. Overall, while this systematic review has limitations, such as potential sample heterogeneity and language bias, the analysis’s strengths, such as its comprehensive evaluation of multiple outcome indicators and consideration of psychological factors, support the efficacy of rTMS in treating NOP. To strengthen the evidence base, further research with larger sample sizes, longer follow-up periods, and the inclusion of studies published in languages other than English would be beneficial.

## 5. Conclusions

Based on the available evidence, it can be concluded that rTMS is a safe and effective therapeutic option for individuals suffering from neuropathic pain (NOP). The analysis suggests that rTMS can improve pain intensity, clinical status, sensory status, and QOL in NOP patients, with a very low to moderate level of evidence. However, it did not show significant improvement in psychological status or sleep quality. It is important to note that further research is needed to better understand the mechanisms of action of rTMS in treating NOP, as well as to investigate its potential for improving psychological and sleep-associated symptoms. Furthermore, future studies with larger sample sizes, longer follow-up periods, and diverse patient populations would help to gain a better understanding of the efficacy and optimal parameters of rTMS in the treatment of NOP.

## Supplementary Material


