# Supplementary material for: Efficacy and safety of repetitive transcranial magnetic stimulation with different frequencies on neuropathic orofacial pain: a systematic literature review and meta-analysis
Source: J Oral Facial Pain Headache. 2024 Jun 12;38(2):48–67. doi: 10.22514/jofph.2024.013 (PMC11810666; doi:10.22514/jofph.2024.013)
Supplement: Supplementary file 1 [file Supplementary-material.docx]

Supplementary material

1. Orofacial neuropathic pain [Mesh]

2. Face Pain [Title/Abstract] OR Pain, Face [Title/Abstract] OR Pain, Facial [Title/Abstract] OR Orofacial Pain [Title/Abstract] OR Pain, Orofacial

[Title/Abstract] OR Neuralgic Facial Pain [Title/Abstract] OR Facial Pain, Neuralgic [Title/Abstract] OR Pain, Neuralgic Facial [Title/Abstract] OR Craniofacial Pain [Title/Abstract] OR Pain, Craniofacial [Title/Abstract] OR Myofacial Pain

[Title/Abstract] OR Pain, Myofacial [Title/Abstract]

3. 1 OR 2

4. Trigeminal Neuralgia [Mesh]

5. Neuralgia, Trigeminal [Title/Abstract] OR Trigeminal Neuralgias [Title/Abstract] OR

Tic Doloureux [Title/Abstract] OR Fothergill Disease [Title/Abstract] OR

Disease, Fothergill [Title/Abstract] OR Trifacial Neuralgia [Title/Abstract] OR

Neuralgia, Trifacial [Title/Abstract] OR Trifacial Neuralgias [Title/Abstract] OR

Tic Douloureux [Title/Abstract] OR Epileptiform Neuralgia [Title/Abstract] OR

Epileptiform Neuralgias [Title/Abstract] OR Neuralgia, Epileptiform [Title/Abstract] OR Secondary Trigeminal Neuralgia [Title/Abstract] OR Neuralgia, Secondary Trigeminal [Title/Abstract] OR Secondary Trigeminal Neuralgias [Title/Abstract] OR

Trigeminal Neuralgia, Secondary [Title/Abstract] OR Trigeminal Neuralgia, Idiopathic [Title/Abstract] OR Idiopathic Trigeminal Neuralgia [Title/Abstract] OR

Idiopathic Trigeminal Neuralgias [Title/Abstract] OR Neuralgia, Idiopathic Trigeminal [Title/Abstract]

6. 4 OR 5

7. Facial Neuralgias [Mesh]

8. Neuralgia, Facial [Title/Abstract] OR Neuralgias, Facial [Title/Abstract] OR Craniofacial Pain Syndromes [Title/Abstract] OR Craniofacial Pain Syndrome [Title/Abstract] OR Pain Syndrome, Craniofacial [Title/Abstract] OR

Pain Syndromes, Craniofacial [Title/Abstract] OR Syndrome, Craniofacial Pain [Title/Abstract] OR Syndromes, Craniofacial Pain [Title/Abstract] OR

Facial Pain Syndromes [Title/Abstract] OR Facial Pain Syndrome [Title/Abstract] OR

Pain Syndrome, Facial [Title/Abstract] OR Pain Syndromes, Facial [Title/Abstract] OR

Syndrome, Facial Pain [Title/Abstract] OR Syndromes, Facial Pain [Title/Abstract] OR

Myofacial Pain Syndromes [Title/Abstract] OR Myofacial Pain Syndrome [Title/Abstract] OR Pain Syndrome, Myofacial [Title/Abstract] OR Pain Syndromes, Myofacial [Title/Abstract] OR Syndrome, Myofacial Pain [Title/Abstract] OR Syndromes, Myofacial Pain [Title/Abstract] OR Sphenopalatine Neuralgia [Title/Abstract] OR Neuralgia, Sphenopalatine [Title/Abstract] OR

Neuralgias, Sphenopalatine [Title/Abstract] OR Sphenopalatine Neuralgias [Title/Abstract]

9. 7 OR 8

10. Glossopharyngeal Nerve Diseases [Mesh]

11. Glossopharyngeal Nerve Disease [Title/Abstract] OR Cranial Nerve IX Disorders [Title/Abstract] OR Ninth Cranial Nerve Diseases [Title/Abstract] OR

Cranial Nerve IX Diseases [Title/Abstract] OR Glossopharyngeal Neuralgia [Title/Abstract] OR Glossopharyngeal Neuralgias [Title/Abstract] OR Neuralgia, Glossopharyngeal [Title/Abstract] OR

Neuralgias, Glossopharyngeal [Title/Abstract] OR Glossopharyngeal Nerve Taste Disorder [Title/Abstract] OR Taste Disorder, Glossopharyngeal Nerve [Title/Abstract] OR Glossopharyngeal Motor Neuropathy [Title/Abstract] OR Glossopharyngeal Motor Neuropathies [Title/Abstract] OR Motor Neuropathies, Glossopharyngeal [Title/Abstract] OR Motor Neuropathy, Glossopharyngeal [Title/Abstract] OR Glossopharyngeal Nerve Sensory Neuropathy [Title/Abstract] OR Sensory Neuropathy, Glossopharyngeal Nerve [Title/Abstract]

12. 10 OR 11

13. Joint, Temporomandibular [Mesh]

14. Joints, Temporomandibular [Title/Abstract] OR Temporomandibular Joints [Title/Abstract] OR TMJ [Title/Abstract] OR Temporomandibular Joint Dysfunction Syndrome [Title/Abstract] OR Myofascial Pain Dysfunction Syndrome, Temporomandibular Joint [Title/Abstract] OR TMJ Syndrome [Title/Abstract] OR Syndrome, TMJ [Title/Abstract] OR Costen’s Syndrome [Title/Abstract] OR Costen Syndrome [Title/Abstract] OR Costens Syndrome [Title/Abstract] OR Syndrome, Costen’s [Title/Abstract] OR Temporomandibular Joint Syndrome [Title/Abstract] OR Joint Syndrome, Temporomandibular [Title/Abstract] OR Syndrome, Temporomandibular Joint [Title/Abstract]

15. 13 OR 14

16. burning mouth syndrome (BMS) [Mesh]

17. Burning Mouth Syndromes [Title/Abstract] OR Mouth Syndrome, Burning [Title/Abstract] OR Mouth Syndromes, Burning [Title/Abstract] OR Syndrome, Burning Mouth [Title/Abstract] OR Syndromes, Burning Mouth [Title/Abstract]

18. 16 OR 17

19. Atypical odontalgia [Title/Abstract] OR Temporomandibular disorders [Title/Abstract] OR Myogenous Temporomandibular Disorders [Title/Abstract] OR Arthrogenous Temporomandibular Disorders [Title/Abstract] OR Neurovascular pain [Title/Abstract] OR Migraine [Title/Abstract] OR Tension-Type Headache [Title/Abstract] OR Trigeminal Autonomic Cephalalgias [Title/Abstract] OR Vascular pain [Title/Abstract] OR Temporal Arteritis [Title/Abstract] OR Central poststroke pain [Title/Abstract] OR

Facial pain attributed to multiple sclerosis [Title/Abstract] OR Anesthesia dolorosa [Title/Abstract]

20. Central neuropathic facial pain [Title/Abstract] OR

Persistent idiopathic facial pain (atypical face pain) [Title/Abstract] OR Burning mouth syndrome [Title/Abstract] OR Tolosa-Hunt Syndrome [Title/Abstract] OR Head or facial pain attributed to herpes zoster [Title/Abstract] OR Other terminal branch neuralgias [Title/Abstract] OR Tension-type headache [Title/Abstract] OR Migraine without or with aura [Title/Abstract] OR Trigeminal neuralgia [Title/Abstract] OR Atypical odontalgia [Title/Abstract] OR TMD/myofacial pain [Title/Abstract] OR cluster headache [Title/Abstract] OR persistent idiopathic facial pain(PIFP) [Title/Abstract]

21. 19 OR 20

22. 3 OR 6 OR 9 OR 12 OR 15 OR 18 OR 21

23. Transcranial Magnetic Stimulation [Mesh]

24. Transcranial Magnetic Stimulation [Title/Abstract] OR Transcranial Magnetic Stimulations [Title/Abstract] OR TMS [Title/Abstract] OR Magnetic Stimulation, Transcranial [Title/Abstract] OR Magnetic Stimulations, Transcranial [Title/Abstract] OR Stimulation, Transcranial Magnetic [Title/Abstract] OR Stimulations, Transcranial Magnetic [Title/Abstract] OR repetitive transcranial magnetic stimulation [Title/Abstract] OR repetitive transcranial magnetic stimulations [Title/Abstract] OR rTMS [Title/Abstract] OR

Transcranial Magnetic Stimulation, Single Pulse [Title/Abstract] OR Transcranial Magnetic Stimulation, Paired Pulse [Title/Abstract] OR Transcranial Magnetic Stimulation, Repetitive [Title/Abstract]

25. 23 OR 24

26. 22 AND 25
